# ﻿Autonomous Reef Monitoring Structures (ARMS) as a tool to uncover neglected marine biodiversity: two new Solenogastres (Mollusca, Aplacophora) from the Gulf of Mexico

**DOI:** 10.3897/zookeys.1221.136385

**Published:** 2024-12-31

**Authors:** M. Carmen Cobo, William J. Farris, Chandler J. Olson, Emily L. McLaughlin, Kevin M. Kocot

**Affiliations:** 1 Department of Invertebrate Zoology, Smithsonian Institution, National Museum of Natural History, Washington, District of Columbia, USA; 2 Department of Biological Sciences, University of Alabama, Tuscaloosa, Alabama, USA; 3 Alabama Museum of Natural History, University of Alabama, Tuscaloosa, Alabama, USA

**Keywords:** Aculifera, biodiversity, conservation, Dondersiidae, mesophotic, Pruvotinidae, sampling methods, taxonomy

## Abstract

Solenogastres is a group of mollusks with evolutionary and ecological importance. Nevertheless, their diversity is underestimated and knowledge about the distribution of the approximately 300 formally described species is limited. Factors that contribute to this include their small size and frequent misidentification by non-specialists. Recent deep-sea explorations have resulted in the collection of numerous specimens through effective methods such as epibenthic sledges. However, this is a costly, labor-intensive, and destructive methodology. In contrast, Autonomous Reef Monitoring Structures (ARMS) offer a novel, non-destructive approach, by providing a substrate for benthic organism colonization. This study is the first to describe Solenogastres collected using ARMS, demonstrating that they are an effective tool for biodiversity assessment and characterizing rare marine invertebrates. Following an integrative taxonomic approach, two new solenogaster species are described: *Dondersiatweedtae* Farris, Olson & Kocot, **sp. nov.** (Dondersiidae) and *Eleutheromeniabullescens* Cobo, **sp. nov.** (Pruvotinidae). The diagnosis of the family Dondersiidae is amended and the necessity of reassessing the validity of the current diagnostic characters for Pruvotinidae, and its classification is emphasized. The two newly described species exhibit distinct external characteristics; *D.tweedtae***sp. nov.** has a striking pink color with a bright yellow dorsal keel and *E.bullescens***sp. nov.** has a unique, discontinuous dorsal keel with nearly spherical protrusions. The presence of cnidocytes in the digestive systems of both species indicate that they feed on cnidarians. It is hypothesized that, like in some nudibranchs, their coloration and body features reflect defensive adaptations related to their diet. This study shows that while habitus alone is typically insufficient for accurate identification in solenogasters, it can sometimes simplify the process. For this, live observations and photographs are essential.

## ﻿Introduction

Solenogastres represents an intriguing group within the phylum Mollusca due to their unique characteristics (worm-shaped body, absence of a shell, reduced foot and mantle cavity) that led to their consideration as early- branching mollusks, and thus important to understanding evolutionary relationships within the phylum (e.g., Salvini-Plawen 1967, 1980, 2003a; Scheltema 1978, 1993, 1996; Sigwart and Sutton 2007; Haszprunar et al. 2008; Kocot et al. 2011; Vinther et al. 2012; Scherholz et al. 2013; Salvini-Plawen and Steiner 2014; Vinther 2014; Yap-Chiongco et al. 2024). The most recent phylogenetic studies supported the placement of Solenogastres with Caudofoveata in a clade (Aplacophora) that with Polyplacophora (chitons) is the sister taxon of all other mollusks (Kocot et al. 2019). Solenogastres exhibit a remarkable ecological versatility, with species described from all latitudes and depths and found in diverse marine habitats: interstitial (e.g., Salvini-Plawen 1986; García-Álvarez et al. 2000; Bergmeier et al. 2016), hydrothermal vents (e.g., Salvini-Plawen 2008; Scheltema 2008), abyssal plains (e.g., Scheltema 1999; Gil-Mansilla et al. 2009; Bergmeier et al. 2017, 2019; Cobo and Kocot 2021) and even the hadal zone (Bergmeier et al. 2019). Some species burrow in the first centimeters of the sediment, while many are epibenthic or epizootic on hydrozoans and corals, and one species was even discovered inside a glass sponge in the Southern Ocean (Kocot et al. 2019). Observations of live specimens are limited, although some classic works (e.g., Pruvot 1890; Heath 1911; Salvini-Plawen 1978) include live observations as well as habitat information, and one work (Scheltema and Jebb 1994) reports on observations of specimens kept alive in an aquarium for several weeks. Nevertheless, most of the life history knowledge of Solenogastres has been inferred through indirect observations of prey remains in the digestive system (mostly cnidarians) and more recently due to contaminated sequences (Okusu and Giribet 2003; Meyer et al. 2010). Bergmeier et al. (2021) exploited resistance of the solenogaster 28S gene to routine PCR amplification to sequence gut contents from species broadly spanning the diversity of the group and found evidence for a high level of dietary specialization within most taxa in the deep-sea. Despite these advances, many questions remain about solenogaster feeding, reproductive behavior, and defense strategies, while the few existing reports on these topics suggest intricate ecological interactions and evolutionary adaptations.

Despite interest in Solenogastres for both evolutionary and ecological reasons, our understanding remains inadequate, starting with an underestimation of the group’s diversity. Just over 300 species have been described to date, but it has been estimated that the true number is tenfold higher (Todt 2013). Likewise, knowledge of species distributions is limited due to sampling bias and many singletons. This lack of knowledge is driven by several factors (reviewed by Todt 2013). Most notably, solenogasters are typically small animals (most measuring ≤ 5 mm) and they are often overlooked or misidentified by non-specialists. In recent years, deep-sea exploration has increased the number of collected solenogasters, mostly due to the efficiency of sampling instruments such as epibenthic sledges (EBS). However, EBS sampling demand significant sorting effort, is a destructive sampling technique, and the preservation of the samples is not always ideal; particularly when the catch is large and must be preserved before sorting. SCUBA diving and remotely operated vehicles (ROVs) are alternative non-destructive methods that, in the case of solenogasters, work well for locating larger specimens and provide live observations and ecological data that would not be possible otherwise. Nevertheless, both are labor-intensive and are unlikely to fully capture the biodiversity of a given site. SCUBA diving is limited by depth and the collection of samples depends on the diver’s eyesight or, in the case of bulk collecting (e.g., sampling reef rubble), how much they can carry. ROV sampling is costly and although it provides valuable images and video, the throughput for specimen collection is low. Autonomous Reef Monitoring Structures (ARMS) represent a novel and standardized approach that offers substrate for benthic organism colonization (www.oceanarms.org). Originally developed during the ‘Census of Marine Life’ to conduct biodiversity assessments and monitoring combining morphological identifications with DNA metabarcoding (Obst et al. 2020), ARMS have proven highly effective for collecting coral reef-associated invertebrates (Zimmerman and Martin 2004). In this study we use an integrative taxonomic approach to describe two new species of Solenogastres collected using ARMS in the Gulf of Mexico as part of the CYCLE project (https://geome-db.org/record/ark:/21547/EBk2): *Dondersiatweedtae* sp. nov. (Dondersiidae) and *Eleutheromeniabullescens* sp. nov. (Pruvotinidae, Eleutheromeniinae). With these two species we increase knowledge of the diversity of Solenogastres in the Gulf of Mexico. To date, only two other species from two different families have been described from the region: *Proneomeniaacuminata* Wirén, 1892 (Proneomeniidae) and *Spengelomeniabathybia* Heath, 1912 (Amphimeniidae).

## ﻿Materials and methods

### ﻿Material examined

**Figure 1.**
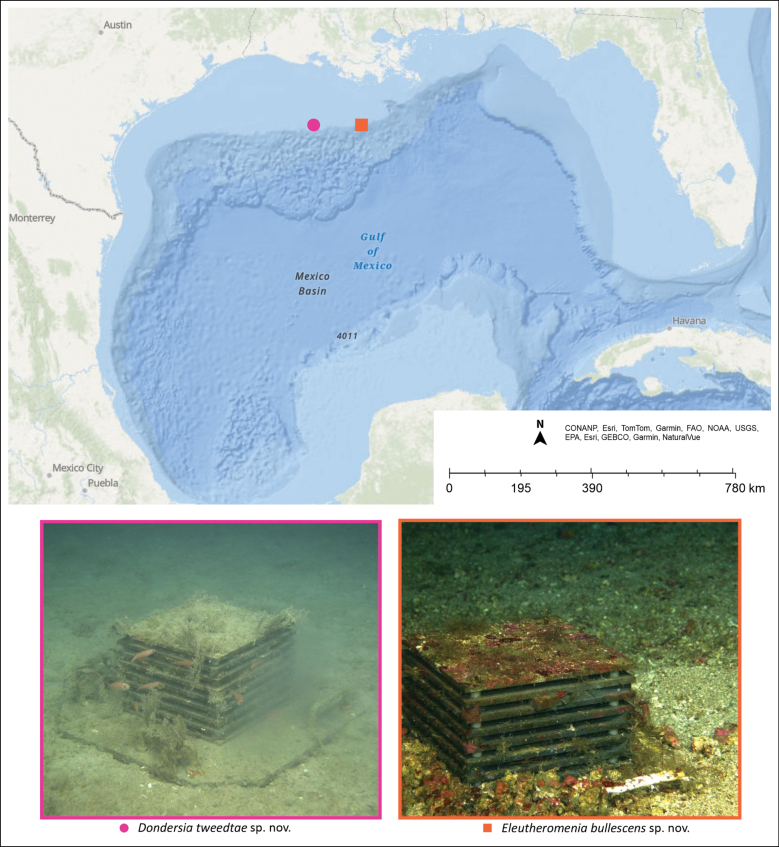
Map with localities where the solenogaster species were found and images of the ARMS.

Three specimens of Solenogastres were collected during the expedition PS21-04 onboard the R/V Point Sur, part of the CYCLE project (https://geome-db.org/record/ark:/21547/EBk2), which aims to assess the connectivity and diversity of mesophotic ecosystems in the Gulf of Mexico. The specimens were collected in two different locations (Fig. 1, Table 1). The specimens were found on Autonomous Reef Monitoring Structures (ARMS) deployed in May 2019 during expedition PS19-25 and recovered in August 2021. All specimens were photographed alive and preserved in 95% ethanol.

**Table 1.** Collection data and final preservation for the examined material (H: Holotype; P: Paratype; Lat: Latitude; Long: Longitude. Depth in meters). Specimens deposited at the Smithsonian National Museum of Natural History.

Museum #	Identification	Type series	Expedition code	Locality	Latitude, Longitude	Depth	Final Preservation
USNM 1718003	*Dondersiatweedtae* sp. nov.	Holotype	CYCLE_2021	Alderdice	28°5'42.18"N, 92°0'20.38"W	82	Serial sections, SEM stub, DNA extraction
USNM 1718004	*Eleutheromeniabullescens* sp. nov.	Holotype	CYCLE_2021	Diaphus	28°5'20.26"N, 90°42'5.06"W	82	Serial sections, SEM stub, DNA extraction
USNM 1718005	*Eleutheromeniabullescens* sp. nov.	Paratype	CYCLE_2021	Diaphus	28°5'20.26"N, 90°42'5.06"W	82	95% ethanol

### ﻿Species descriptions

#### ﻿Habitus and hard parts

Specimens were sorted into two morphospecies based on the study of habitus (coloration, sclerite appearance, body protrusions, body shape). Preserved specimens were observed, photographed using an Olympus SZ40 dissecting microscope with an Olympus DP71 digital camera, and measured. The length of each specimen in lateral view was measured along the axial midline; the dorso-ventral height was also measured in lateral view. In addition, after decalcification (see methodology below) one of the specimens (USNM 1718004) was photographed using an Olympus DSX100 microscope to observe details of the dorsal body protrusions. Photographs of the fixed material were compared with field photographs for a proper characterization of the external aspect. Two of the specimens (one of each morphospecies) were cut into three parts. The medial body region was air-dried and imaged (uncoated) using a Phenom Pro scanning electron microscope (SEM) under low vacuum with a low accelerating voltage (5–10 kV) to study the sclerites. Subsequently, dried tissue samples were put directly into Omega Bio-tek E.Z.N.A. MicroElute kit tissue lysis (TL) buffer and frozen at -80 °C for later DNA extraction. The anterior and posterior regions were retained in 95% ethanol until they were used for histology.

#### ﻿Histology

To analyze internal anatomy, the anterior and posterior body regions of two of the specimens (one of each morphospecies; Table 1) were decalcified with EDTA solution (2 ml of distilled water; 1 ml of 10% formalin; and 2 ml of 0.5M EDTA) overnight, dehydrated with a graded ethanol series (20 min for each soak: 70% - 90% - 90% - 95% - 95% - 100% - 100% ethanol) followed by a xylene soak (until the tissue was translucent; ~15 min), embedded in paraffin (Leica Paraplast Regular) following three soaks in fresh paraffin for 1 h each, cut in 5 μm serial transverse sections using a Leica RM2235 rotary microtome and a Reichert-Jung 820 II Histocut Microtome, and stained with Mallory’s trichrome stain. The staining protocol followed Gil-Mansilla et al. (2008) except the xylene step was reduced to one soak of < 15 min (just until tissues were translucent), the embedding in paraffin step to two hours instead of three, and the second stain was performed for 20 min. Histological sections of SH20364 were imaged using an Olympus BX53 compound microscope with an SC50 digital camera. Histological sections of SH20192-A and SH20192-B were imaged using an Olympus BX63F compound microscope. A manual reconstruction was made for each species following the structures under the microscope. The manual reconstructions were then digitalized using Corel Draw Standard 2021.

### ﻿DNA barcoding and phylogenetic analysis

#### ﻿DNA barcoding

DNA was extracted from the mid-body tissue used for SEM with the Omega Bio-tek E.Z.N.A. MicroElute kit following the manufacturer’s protocol. PCR amplification of a fragment of the mitochondrial 16S rDNA (16S), cytochrome c oxidase subunit I (COI) and cytochrome B (CytB) were performed using Hot Start Taq 2X Master Mix (VWR) following the manufacturer’s instructions. For 16S, the solenogaster-specific primers 16Soleno-r and 16Soleno-f (Bergmeier et al. 2017) were used with the following cycling parameters: 1 min at 94 °C, (15 s at 94 °C, 30 s at 50 °C, 1 min at 72 °C) × 35 cycles, 7 min 68 °C and finally cooling at 10 °C. For COI, the primers LCO_Apl (TTTCTACTAAYCATAARGATATTGG) and HCO 2198 (Folmer et al. 1994) were used with the following cycling parameters: 1 min at 94 °C, (15 s at 94 °C, 30 s at 52 °C, 1 min at 72 °C) × 30 cycles, 7 min 68 °C and finally cooling at 10 °C. For CytB, the primers 424F and 876R (Boore and Brown 1994) were used with the following cycling parameters: 1 min at 94 °C, (15 s at 94 °C, 30 s at 47 °C, 1 min at 72 °C) × 40 cycles, 7 min 68 °C and finally cooling at 10 °C. PCR success was determined with gel electrophoresis using 1X SB buffer at 120 volts for 20 min. Products were directly purified either using the Omega Bio-tek E.Z.N.A. Cycle Pure Quick kit or using AMPure SPRI magnetic beads for a one-sided size selection using .95× beads and were eluted in 25 µl of elution buffer. The concentration of the purified PCR products was measured with a Qubit 3.0 Fluorometer using dsDNA HS reagents (Invitrogen). Purified PCR products were sent to GeneWiz for bidirectional Sanger sequencing. Sequencing was performed using the premix option with 10 µl of PCR product and 5 µL of 5 µM primer for each reaction. Successful DNA sequences were assembled into contigs, inspected, and manually edited for quality, if needed, using Geneious Prime 2024. Finally, a BLAST search against the NCBI Nucleotide database was performed to check for any contaminated sequences. All newly generated sequences have been made publicly available via NCBI (Table 2).

**Table 2.** Accession numbers of the sequences used for the phylogenetic analysis (16S and COI) and of the obtained sequences for the new species.

Species	COI	16S	CytB	Reference
*Alexandromeniacrassa* Odhner, 1920	MG855758	MG855855		Mikkelsen et al. 2019
*Anameniagorgonophila* (Kowalevsky, 1880)	OQ597876	OQ600030		Cobo et al. 2023
*Apodomeniaenigmatica* Kocot, Todt, Mikkelsen & Halanych, 2019	MK404653	PQ226473		Kocot et al. 2019
Chaetodermanitidulum Lovén, 1844	AY377726	AY377612		Okusu et al. 2003
*Dondersiafestiva Hubrecht*, *1888*	OR458916	OR456222		Cobo et al. 2024
*Dondersiatweedtae* sp. nov.	PQ246886	PQ249005	PQ241521	Present study
*Dorymeniatricarinata* (Thiele, 1913)	OQ600547	OQ618431		Todt and Kocot 2014; Cobo et al. 2023
*Eleutheromeniabullescens* sp. nov.	PQ246885	PQ249006	PQ241520	Present study
*Eleutheromeniasierra* (Pruvot, 1890)	OR458913	OR456216		Cobo et al. 2024
*Epimeniababai* Salvini-Plawen, 1997	AY377724	AY377616		Okusu et al. 2003
*Falcidenssagittiferus* Salvini-Plawen, 1968	MG855748	MG855834		Mikkelsen et al, 2019
*Gymnomeniapellucida* Odhner, 1920	OQ600550	OQ618433		Cobo et al. 2023
*Helluoherpiaaegiri* Handl & Büchinger, 1996	PQ222747	PQ226470		Present study
*Hypomeniasanjuanensis* Kocot & Todt, 2014	OQ600549	OQ618434		Cobo et al. 2023
*Kruppomeniagenslerae* Ostermair et al. 2018	MN531184	MG603271		Bergmeier et al. 2019; Ostermair et al. 2018
*Macellomeniaschanderi* Kocot & Todt, 2014	KJ568516	PQ226471		Kocot et al. 2017
*Micromeniafodiens* (Schwabl, 1955)	PQ222750	n/a		Kocot et al. 2019
*Nematomeniabanyulensis* (Pruvot, 1890)	OR458911	OR456215		Cobo et al. 2024
*Neomeniamegatrapezata* Salvini-Plawen & Paar-Gausch, 2004	PQ222749	PQ226472		Present study
*Proneomeniacustodiens* Todt & Kocot, 2014	KJ568518	OQ618430		Cobo et al. 2023; Kocot and Todt 2014
*Proneomeniasluiteri* Hubrecht, 1880	KJ568517	OQ618429		Todt and Kocot 2014; Cobo et al. 2023
Pruvotiacf.sopita (Pruvot, 1891)	OR458908	OR456214		Cobo et al. 2024
*Pruvotinaimpexa* (Pruvot, 1890)	OR458907	n/a		Cobo et al. 2024
*Scutopusventrolineatus* Salvini-Plawen, 1968	MG855751	MG855840		Mikkelsen et al. 2019
*Simrothiellamargaritacea* (Koren & Danielssen, 1877)	OQ600548	OQ618432		Cobo et al. 2023
*Stylomeniasulcodoryata* Handl & Salvini-Plawen, 2001	OR452313	PQ226469		Cobo et al. 2024; present study
*Tegulaherpiatasmanica* Salvini-Plawen, 1988	PQ222746	PQ226468		Yap-Chiongco et al. 2024
*Unciherpiahirsuta* Urgorri & Salvini-Plawen, 2001	OQ597875	OQ600031		Cobo et al. 2023
*Wireniaargentea* Odhner, 1920	MG855759	MG855856		Mikkelsen et al. 2019

### ﻿Phylogenetic analysis

To confirm our morphology-based identifications, a phylogenetic analysis was performed based on COI and 16S sequences. In addition to data from the new species, sequences broadly spanning the diversity of Solenogastres were obtained from NCBI based on the results of Kocot et al. (2019) as well as available sequences of close relatives of the new described species (Table 2). The caudofoveates *Chaetodermanitidulum* Lovén, 1844. *Scutopusventrolineatus* Salvini-Plawen, 1968, and *Falcidenssagittiferus* Salvini-Plawen, 1968 were used as the outgroup. Sequences were aligned with MAFFT v. 7 (Katoh et al. 2002), and the resulting alignments were manually refined to ensure protein-coding sequences (COI) were in the correct open reading frame prior to concatenation with Mesquite 3.81. (Maddison and Maddison 2023). A phylogenetic analysis was conducted on the resulting alignment using maximum likelihood in IQ-TREE 2 (Minh et al. 2020) with the best-fitting model of nucleotide substitution for each partition and 1000 rapid bootstraps. For 16S, the model used was GTR+F+I+G4. COI was additionally partitioned by codon position. Codon position 1 used GTR+F+R3, position 2 used TN+F+R3, and position 3 used TIM2+F+I+G4.

## ﻿Results

### ﻿Species descriptions

**Order Pholidoskepia Salvini-Plawen, 1978**

**Family Dondersiidae Simroth, 1893**

#### ﻿Genus Dondersia

Taxon classificationAnimaliaPholidoskepiaDondersiidae

Hubrecth, 1888

D3E7BC1B-92F7-5484-B63F-6BFA575A03F8

##### Type species.

*Dondersiafestiva* Hubrecht, 1888, by monotypy. Type locality. Mediterranean Sea (northern Gulf of Naples); 60 m.

#### ﻿ Dondersia tweedtae

Taxon classificationAnimaliaPholidoskepiaDondersiidae

Farris, Olson & Kocot sp. nov.

0E60E8F9-1A29-568E-BE9D-3CBC4015F750

https://zoobank.org/FE62C5A8-71BD-40C4-9E76-288CD3D93AE6

##### Examined material.

***Holotype***: SH20364 (USNM 1718003). Gulf of Mexico. 28°5'42.18"N, 92°0'20.38"W. 82 m depth. Serial sections (23 slides), light microscopy preparations of the sclerites (two slides, sclerite from mid-body); SEM stub with sclerites; COI, 16S, and CytB sequences (NCBI PQ246886, PQ249005, and PQ241521, respectively).

##### Derivatio nominis.

Named after Dr. Sarah Tweedt who provided us with the material and for her outstanding work studying invertebrate biodiversity using ARMS; *tweedtae* is feminine in the genitive.

##### Diagnosis.

Elongate animal (~ 14 mm), bright pink with a yellow dorsal keel bearing 17 distinct lobes. Smooth, scaled appearance with three distinct scale-like sclerite types. Large anterior pedal glands. Atrium with about 14 trilobed papillae. Mouth separated from the atrium. Ventrolateral foregut glands of type A. Monoserial radula with two denticles joined at their apex. Midgut with a short dorsal caecum, without lateral constrictions. With five dorsoterminal sensory organs. Without accessory copulatory structures.

##### Description.

Description based on the holotype. Reconstruction of the internal anatomy based on manual reconstruction of the histological sections (Fig. 8A, A’).

**Figure 2.**
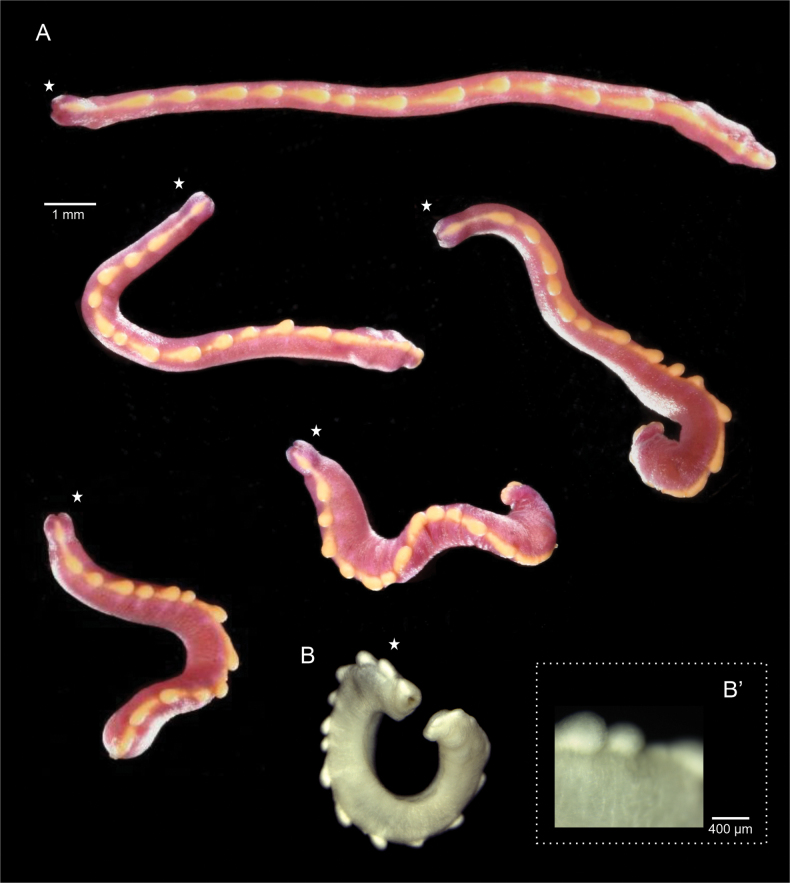
Habitus of *Dondersiatweedtae* sp. nov. **A** field photographs of the holotype showing the contractions and extension range (usnm 1718003) **B** photograph of the holotype preserved in ethanol **B**’ detail of the lobes of the dorsal keel. Star indicates the anterior end of the animal.

***Habitus*.** Long animal (14 mm, 0.55 mm wide in the midbody), pink color aside from the bright yellow, continuous dorsal keel composed of 17 serially arranged lobes (Fig. 2A). Body with shiny and slightly scaly appearance Coloration fades to off-white in 95% ethanol (Fig. 2B). Animal extends and contracts the body significantly; it varies in length, ranging from ~ 6–14 mm, and its width spans from 0.8–2 mm. (Fig. 2A). Tapered anterior. Posterior with a slight finger-like projection. Pedal groove, mantle cavity and mouth apertures visible externally (Fig. 2B).

**Figure 3.**
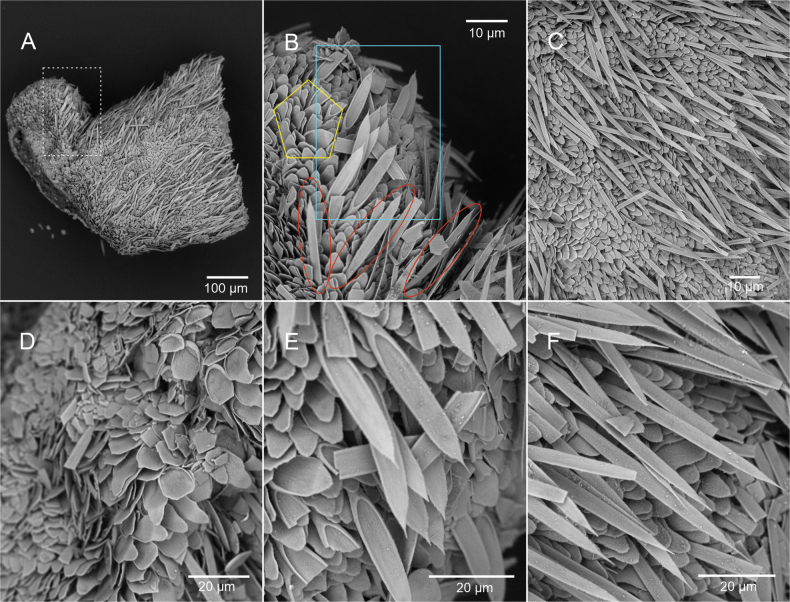
SEM images of the sclerites of *Dondersiatweedtae* sp. nov. **A** general SEM image of the dorsal and mid body **B** corresponds with the white square in **A** oval-shaped scales (yellow pentagon), lanceolate scales (red ovals) and pedunculated leaf-shaped scales (blue square) **C** lanceolate scales among oval-shaped scales **D** detail of the layer of oval-shaped scales **E** pedunculated leaf-shaped scales among oval-shaped scales **F** detail of the lanceolate scales. (Images of the holotype: USNM 1718003).

***Mantle*.** Thin epidermis (17.54–36.57 μm thick, thickness decreases to ~ 10 μm in areas of the posterior end of the body) without epidermal papillae. Three types of sclerites as scales inserted in one layer (Fig. 3): 1) Oval-shaped scales, relatively small (14–17.61 μm long, 7.69–9.79 μm wide) with a proximal rim and an elongated distal end (Fig. 3B, D), most common type, which forms a base layer across the entire body; 2) Lanceolate scales, long and narrow (38.57–39.75 μm long, 5.28–6.86 μm wide) with an acute distal end (Fig. 3B, C, F), distributed intermittently among the oval-shaped sclerites and are less abundant and shorter on the lobes of the dorsal keel; and 3) Pedunculated paddle-like (i.e., oar-shaped scales (Fig. 3B, E; 38.57–39.75 μm long, 5.28–6.86 μm wide), ‘paddle’ portion with a proximal rim, distal edge finely serrated ending in an acuminate point. Paddle-like sclerites inserted in the cuticle amongst the oval-shaped scales, found in the dorsal keel. Scales of the pedal groove not observed.

**Figure 4.**
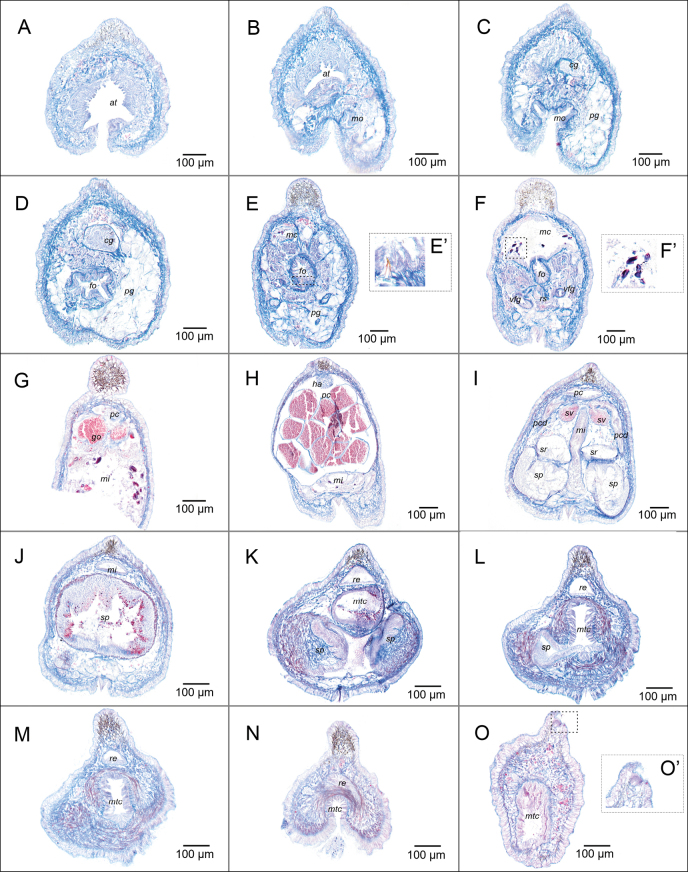
Serial section of *Dondersiatweedtae* sp. nov. **A–F** anterior region **A** atrium **B** atrium and mouth **C** opening of the mouth and cerebral ganglion **D** pre-radular region of the foregut and cerebral ganglion **E** radular region of the foregut and midgut caecum **E**’ detail of the radula **F** radular region of the foregut: radular sac, ventrolateral foregut glands and midgut caecum **F**’ detail of the cnidocytes in the midgut caecum **G** mid-posterior region of the body **H–O** posterior region **H** pericardium bearing the heart and reproductive cells **I** paired origin of the spawning ducts, termination of the pericardium **J** pericardium bearing the heart and reproductive cells **K–M** evolution of the fused region of the spawning ducts **N** opening of the mantle cavity **O** posterior pouch of the mantle cavity and dorsoterminal sensory organ. Abbreviations: at – atrium; cg – cerebral ganglia; fo – foregut; mc – midgut caecum; mi – midgut; mo – mouth; mtc – mantle cavity; pcd – pericardioducts; pg – pedal gland; re – rectum; rs – radular sac; sp – spawning duct; sv – seminal vesicle; sr – seminal receptacles; vfg – ventrolateral foregut glands. (Images of the holotype: USNM 1718003).

***Pedal groove and mantle cavity*.** Pedal pit (100 μm long, 165 μm wide, 100–140 μm high) located posteriorly to the mouth. Pedal glands very large, reaching the dorsal part of the body, surrounding the foregut (Fig. 4B–E). Well-defined pedal groove with a single triangular fold (30–60 μm wide, 40–65 μm tall). Mantle cavity (170 μm long, 320 μm high in the middle region) opens ventrally, with posterior pouch (Fig. 4O). Without respiratory folds, walls of the mantle cavity appear slightly folded and ciliated (Fig. 4N).

***Nervous system and sensory organs*.** Cerebral ganglion circular to oval shape in cross section (85 μm long, 50–180 μm wide, 57–110 μm high; Fig. 4C, D). Atrium (182 μm long, 120–200 μm wide 100 μm–260 μm high) opens ventrally with about 24 atrial papillae distally trilobed (27.5–52.5 μm long, 2.5–7.5 μm wide). Five dorsoterminal sensory organs observed both externally and in the serial sections (Fig. 4O).

***Digestive system*.** Mouth opens ventrally, separated from the atrium (Figs 4B, C, 8A). Foregut rounded and narrow (50–70 μm diameter), surrounded by a glandular epithelium and a thin muscular layer. Monoserial radula composed of a broad, non-serrated base (~ 20–25 μm wide, 5–10 μm high) and two long and narrow denticles that join at their apex (20–25 μm high, 2.5–5 μm wide; Fig. 4E, E’). Fragments of what seems to be two small lateral teeth observed in the edges of the base (Fig. 4E’). Radular sac extends posteriorly (Fig. 4F; 35 μm long, and up to 45 μm wide, 75 μm high). Ventrolateral foregut glands of type A (García-Álvarez and Salvini-Plawen 2007) join the foregut via a common opening (Fig. 4E). Esophagus (95 μm long, 35–40 μm in diameter) forms a sphincter as it joins the midgut centrally (Fig. 8A). Midgut with a single dorso-anterior caecum (Fig. 8A) that contains cnidocytes (Fig. 4F, F’), also found in the midgut. Rectum (80–150 μm in diameter) discharges dorsally into the mantle cavity.

***Gonopericardial system*.** Mature animal. Large pericardium (640 μm long, 100 to 530 μm diameter; significantly narrow in its posterior region: 70 μm diameter) (Fig. 8A’), closely associated with gonads, separated only by a thin tissue layer without defined gonoducts (Fig. 4G, H). Heart attached to the dorsal wall of the pericardium (Fig. 4H). Short pericardioducts (60 μm long, 10–20 μm diameter) that connect to the very posterior end of the pericardium and with the spawning ducts in their origin (Fig. 4I). One seminal vesicle attached to each pericardioduct (Fig. 4I). Fused region of the spawning ducts (400 μm long, up to 320 μm in diameter) about double the length of the paired region (Fig. 4J). Spawning ducts terminate into the antero-dorsal region of the mantle cavity (Fig. 8A’) as a single duct (Fig. 4M), with two glandular lateral pouches in its posterior region (Figs 4K, L, 8A’).

***Anatomy of the dorsal keel*.** Continuous cuticular dorsal keel made up of 17 lobes covered by cuticle and sclerites. The serially arranged lobes are connected as can be seen externally through the yellow coloration in the living specimen (Fig. 2A). Lateral view of the animal shows how the region between lobes is a bit elevated and thus constitutes a continuous keel. Serial sections show stained dark brown cells (with Mallory’s Trichrome) contained in the lobes. This stained content is concentrated in the cavity of the lobules, but also continues into the cuticle. The fact that this can be seen in all the series of sections is an additional proof to the morphological continuity of the keel (Fig. 4).

##### Comparisons.

Considering the traditional classification of Solenogastres (*sensu*Salvini-Plawen 1978), the order Pholidoskepia is characterized by a thin cuticle and sclerites as scales. Some authors have identified issues within this order calling for a taxonomic revision (Scheltema 1999; Scheltema and Schander 2000; Scheltema et al. 2012; Bergmeier et al. 2016, 2019; Yap-Chiongco et al. 2024). Nevertheless, the grouping of Pholidoskepia sensu stricto (Yap-Chiongco et al. 2024) is well-supported by the mentioned mantle characteristics and molecular data. Thus, we follow the traditional classification here. Within Pholidoskepia, the mantle sclerites, radula, and type of ventrolateral foregut glands, as well as some posterior organs, are important characters used to classify specimens into a family (García-Álvarez and Salvini-Plawen 2007). Particularly important for the identification of Dondersiidae species is the types of sclerites (Scheltema et al. 2012; Cobo and Kocot 2021). The sclerites of the specimen studied here can be compared to those described previously for species of *Dondersia*, especially with those of the type species: *Dondersiafestiva* (Hubrecht 1888: fig. 13-2a; Scheltema et al. 2012: figs 1–3). This, with the structure of the radula, justify the classification of the new species within this genus. Moreover, our phylogenetic analysis also supports this classification (see below). The coloration of living specimens is unknown for most solenogasters as most of the species have been described based on preserved material. Within *Dondersia*, two described species are known to have bright colorations: *D.festiva* (bright purple) and *D.annulata* Nierstrasz, 1902 (hot pink with white stripes). Despite similarities, there are clear differences between *Dondersiatweedtae* sp. nov. and these two species and the remaining species of the genus (reviewed in Cobo and Kocot 2021). Particularly, the combination of pink and yellow coloration, along with the dorsal cuticular lobes, is exclusive to *D.tweedtae* sp. nov. Moreover, this constitutes the first Dondersiidae from the Gulf of Mexico (Table 3).

**Table 3.** Species of the families Dondersiidae Simroth, 1893 and Pruvotinidae Heath, 1911 with their know distributions.

Subfamily	Genus	Species	Distribution	Depth (m)
**Dondersiidae** Simroth, 1893	***Dondersia*** Hubrecht, 1888	*Dondersia* (?) *todtae*Klink et al., 2015	Azores (North Atlantic)	26
		*Dondersianamibiensis* Scheltema, Schander & Kocot, 2012	Namibia (South Atlantic)	619–1007
		*Dondersiaincali* (Scheltema, 1999)	West European Basin (North Atlantic)	2091
		*Dondersiacnidevorans* Salvini-Plawen, 1978	Ross Sea (Southern Ocean)	659–714
		*Dondersialaminata* Salvini-Plawen, 1978	Graham Land, Bransfield Strait (Southern Ocean)	311–426
		*Dondersiastylastericola* Salvini-Plawen, 1978	South Shetland Islands (Southern Ocean)	300
		*Dondersiaannulata* Nierstrasz, 1902	Bima, Sumbawa (Indo-Pacific)	55
		*Dondersiafestiva* Hubrecht, 1888	Gulf of Naples. Corsica (Mediterranean Sea)	60
		Dondersia?foraminosa Cobo & Kocot, 2021	Brazil Basin (South Atlantic)	4484.7 - 4503
		***Dondersiatweedtae* sp. nov.**	**Gulf of Mexico**	**82**
	***Heathia*** Thiele, 1913	*Heathiaporosa* (Heath, 1911)	San Diego, California (Northeast Pacific)	920–990
	***Helluoherpia*** Handl & Büchinger, 1996	*Helluoherpiavieiralaneroi* Cobo & Kocot, 2021	Brazil Basin (South Atlantic)	4484.7-4503
		*Helluoherpiaaegiri* Handl & Büchinger, 1996	Herdlafjord, Bergen. (Norwegian Sea)	185–250
	***Ichthyomenia*** Pilsbry, 1898	*Ichthyomeniaichthyodes* (Pruvot, 1890)	Rousillon, France (Mediterranean Se)	80
	***Inopinatamenia*** Cobo & Kocot, 2021	*Inopinatameniacalamitosa* Cobo & Kocot, 2021	Brazil Basin (South Atlantic)	4484.7-4503
	***Lyratoherpia*** Salvini-Plawen, 1978	*Lyratoherpiabracteata* Salvini-Plawen, 1978	South Sandwich Islands (Southern Ocean)	148–201
		*Lyratoherpiacarinata* Salvini-Plawen, 1978	Ross Sea (Southern Ocean)	344–714
		*Lyratoherpiacalifornica* (Heath, 1911)	San Diego, California (Northeast Pacific)	38–46
	***Micromenia*** Leloup, 1948	*Micromeniaamphiatlantica* Cobo & Kocot, 2020	Brazil, Angola, Guinea Basins (South Atlantic)	5433–5460
		*Micromeniasubrubra* Salvini-Plawen, 2003	Malta (Mediterranean Sea)	140
		*Micromeniasimplex* Leloup, 1948	Hope Island, Barents Sea (Artic)	48
		*Micromeniafodiens* (Schwabl, 1955)	Gullmarfjord, Sweeden (North Atlantic)	40
	***Nematomenia*** Pruvot, 1890	*Nematomeniaglacialis* Thiele, 1913	Gauss Station, Davis Sea (Southern Ocean)	385
		*Nematomeniaincirrata* Salvini-Plawen, 1978	South Orkney Islands (Southern Ocean)	298–302
		*Nematomeniaprotecta* Thiele, 1913	Gauss Station, David Sea (Southern Ocean)	385
		*Nematomeniaptyalosa* Salvini-Plawen, 1978	Sandwich Islands (Antarctica) to Tiera de Fuego	148–210
		*Nematomeniasquamosa* Thiele, 1913	Gauss Station, Davis Sea (Southern Ocean)	385
		*Nematomeniategulata* Salvini-Plawen, 1978	South Sandwich Islands (Southern Ocean)	148–201
		Nematomenia?guineana Cobo & Kocot, 2021	Guinea Basin (South Atlantic)	5142
		*Nematomeniabrasiliensis* Cobo & Kocot, 2021	Brazil Basin (South Atlantic)	4500
		*Nematomeniadivae* Cobo & Kocot, 2021	Guinea Basin (South Atlantic)	5144
		*Nematomeniaplatypoda* (Heath, 1911)	Aleutian Islands, Bering Sea (North Pacific)	880
		*Nematomeniabanyulensis* (Pruvot, 1890)	Dalmatia (Mediterranean Sea) to Trondheimsfjord (Norwegian Sea)	45–300
**Dondersiidae** Simroth, 1893	***Nematomenia*** Pruvot, 1890	*Nematomeniacorallophila* (Kowalevsky, 1881)	Algeria (Mediterranean Sea)	73–183
		*Nematomeniaflavens* (Pruvot, 1890)	Banyuls, Costa Brava, Corsica (Mediterranean Sea) to Shetland Islands (North Sea)	45–167
		*Nematomeniaarctica* Thiele, 1913	Spitzbergen, Svalbard Archipelago (Artic)	
	***Squamatoherpia*** Büchinger & Handl, 1996	*Squamatoherpiatricuspidata* Büchinger & Handl, 1996	Bergen (Norwegian Sea)	250
	***Stylomenia*** Pruvot, 1899	*Stylomeniasalvatori* Pruvot, 1899	Banyuls sur Mer (Mediterranean Sea)	Littoral
		*Stylomeniasulcodoryata* Handl & Salvini-Plawen, 2001	Bergen (Norwegian Sea)	185
Pruvotininae Heath, 1911	***Pruvotina*** Cockerell, 1903	*Pruvotinacryophila* (Pelseneer, 1901)	Bellinghausen Sea (Southern Ocean)	342–550
		*Pruvotinagauszi* Salvini-Plawen, 1978	Gauss Station, David Sea (Southern Ocean)	385
		*Pruvotinalongispinosa* Salvini-Plawen, 1978	Drake Strait, South Sandwich Islands (Southern Ocean)	64–220/3890?
		*Pruvotinamanifesta* Zamarro, García-Álvarez & Ugorri, 2013	Antarctic Peninsula (Southern Ocean)	254
		*Pruvotinapallioglandulata* Salvini-Plawen, 1978	South Shetland Islands (Southern Ocean)	210–220
		*Pruvotinapraegnans* Salvini-Plawen, 1978	South Sandwich Islands (Southern Ocean)	148–220
		*Pruvotinaprovidens* Thiele, 1913	Gauss Station, David Sea (Southern Ocean)	385
		*Pruvotinauniperata* Salvini-Plawen, 1978	Ross Sea (Southern Ocean)	210–2306
		*Pruvotinaimpexa* (Pruvot, 1890)	Banyuls sur Mer, Corsica (Mediterranean Sea)	60–80
		*Pruvotinaartabara* Zamarro, García-Álvarez & Ugorri, 2013	NW Iberian Peninsula (North Atlantic)	1132–1191
		*Pruvotinamegathecata* Salvini-Plawen, 1978	Tierra de Fuego (South Pacific)	118–903
		*Pruvotinapeniculata* Salvini-Plawen, 1978	Tierra de Fuego (South Pacific)	119–549
		*Pruvotinabathyalis* Pedrouzo, García-Álvarez & Urgorri, 2022	NW Iberian Peninsula (North Atlantic)	566–581
		*Pruvotinaglandulosa* Pedrouzo, García-Álvarez & Urgorri, 2022	NW Iberian Peninsula (North Atlantic)	980–2516
		*Pruvotinaharpagone* Pedrouzo, García-Álvarez & Urgorri, 2022	NW Iberian Peninsula (North Atlantic)	709–728
		*Pruvotinazamarroae* Pedrouzo, García-Álvarez & Urgorri, 2022	NW Iberian Peninsula (North Atlantic)	600
	***Pararrhopalia*** Simroth, 1893	*Pararrhopaliafasciata* Salvini-Plawen, 1978	South Sandwich Islands (Southern Ocean)	220–240
		*Pararrhopaliapruvoti* Simroth, 1893	Banyuls sur Mer (Mediterranean Sea)	80–150
		*Pararrhopaliaoscari* Pedrouzo & Urgorri, 2022	NW Iberian Peninsula (North Atlantic)	438–459
	***Labidoherpia*** Thiele, 1903	*Labidoherpiaspinosa* (Thiele, 1913)	Gauss Station, (Southern Ocean)	385
		*Labidoherpialucu*s Pedrouzo, García-Álvarez & Urgorri, 2022	NW Iberian Peninsula (North Atlantic)	616
		*Labidoherpiavitucoi* Pedrouzo & García-Álvarez, 2022	NW Iberian Peninsula (North Atlantic)	438–459
Eleutheromeniinae Salvini-Plawen, 1978	***Eleutheromenia*** Salvini-Plawen, 1967	*Eleutheromeniasierra* (Pruvot, 1890)	Mediterranean Sea to Norway	40–610
		*Eleutheromeniaantarctica* Salvini-Plawen, 1978	Ross Sea (Southern Ocean)	342–714
		***Eleutheromeniabullescens* sp. nov.**	**Gulf of Mexico**	**82**
	***Luitfriedia*** García-Álvarez & Urgorri, 2001	*Luitfriediaminuta* García-Álvarez & Urgorri, 2001	NW Iberian Peninsula (North Atlantic)	760–769
Lophomeniinae Salvini-Plawen, 1978	***Lophomenia*** Heath, 1911	*Lophomeniaspiralis* Heath, 1911	Nilhau Islands, Hawaii (East Pacific)	100–1200
		*Lophomeniadorsocaeca* Gil-Mansilla, García-Álvarez & Urgorri, 2011	Angola Basin (South Atlantic)	5390–5415
Lophomeniinae Salvini-Plawen, 1978	***Hypomenia*** van Lummel, 1930	*Hypomeniasanjuanensis* Kocot & Todt, 2014	San Juan Channel (Northeast Pacific)	59
		*Hypomenianierstraszi* Van Lummel, 1930	Gulf of Naples (Mediterranean Sea)	150–200
	***Metamenia*** Thiele, 1913	*Metameniaintermedia* Thiele, 1913	Gauss Station, David Sea (Southern Ocean)	293–385
		*Metameniatriglandulata* Salvini-Plawen, 1978	Ross Sea (Southern Ocean)	342–1610
Halomeniinae Salvini-Plawen, 1978	***Halomenia*** Heath, 1911	*Halomeniagravida* Heath, 1911	Kuril Islands (Northwest Pacific)	420
	***Forcepimenia*** Salvini-Plawen, 1969	*Forcepimeniaprotecta* Salvini-Plawen, 1969	Red Sea and Gulf of Aden	30
Unciherpiinae Garcia-Alvarez, Urgorri & Salvini-Plawen, 2001	***Uncimenia*** Nierstrasz, 1903	*Uncimenianeapolitana* Nierstrasz, 1903	Gulf of Naples (Mediterranean Sea)	70
	***Sialoherpia*** Salvini-Plawen, 1978	*Sialoherpiaaculeitecta* Salvini-Plawen, 1978	Drake Strait	2782–2827
Scheltemaiinae Pedrouzo, Garcia-Alvarez & Urgorri, 2022	***Scheltemaia*** Salvini-Plawen, 2003	*Scheltemaiamimus* (Scheltema & Schander, 2000)	Bass Strait (Tasmania)	140
		*Scheltemaiabassensis* (Scheltema & Schander, 2000)	Bass Strait (Tasmania)	70

Since this new species bears a cuticular keel, the diagnosis of the family, which states the absence of dorsal keel (Scheltema et al. 2012; Cobo and Kocot 2021), needs to be amended to: “elongate body with or without keel. Anterior end tapered, posterior end with a finger-like projection. Leaf-shaped scales as most abundant type of sclerite, with oar-shaped (= pallet-shaped) or laminar scales scattered between them. With or without common atrio-buccal cavity. Monoserial radula; teeth with four denticles; two central denticles fused and curved distally; two lateral, curved denticles arising from a rounded base. With or without dorsoterminal sensory organs. With or without copulatory stylets. Without respiratory folds. With seminal vesicles and with or without seminal receptacles.”

###### ﻿Order “Cavibelonia” Salvini-Plawen, 1978

**Family Pruvotinidae Heath, 1911**

**Subfamily Eleutheromeniinae Salvini-Plawen, 1978**

#### ﻿Genus Eleutheromenia

Taxon classificationAnimaliaCavibeloniaPruvotinidae

Salvini-Plawen, 1967

CE31827F-6366-5C11-B81F-7C67EBF7F11E

##### Type species.

*Parameniasierra* Pruvot, 1890, by monotypy. Type locality. Costa Brava (Mediterranean Sea); 80 m. Type material missing (García-Álvarez and Salvini-Plawen 2007).

#### ﻿ Eleutheromenia bullescens

Taxon classificationAnimaliaCavibeloniaPruvotinidae

Cobo sp. nov.

F701C8FF-1D33-55DD-A99F-706E9EE079B3

https://zoobank.org/B6796295-A389-4B86-80CE-CA4C61C1A5C1

##### Examined material.

***Holotype***: SH20192-A (USNM 1718004) Gulf of Mexico. CYCLE 2021 event ID CYCLE_2021_ARMS_01_DIAback: 28.088295, -90.701405. 82 m depth. Serial sections (16 slides 5 µm), light microscopy preparation of the sclerites (1 slide); SEM stub with sclerites; COI, 16S, and CytB Sequences (NCBI PQ246885, PQ249006, and PQ241520, respectively). ***Paratype*** SH20192-B (USNM 1718005) Gulf of Mexico. 28.088295, -90.701405. 82 m depth. Animal preserved in 95% ethanol.

##### Derivatio nominis.

From Latin *bullesco*, *bullescis*, *bullescere*; meaning “to bubble” or “to form bubbles” due to the aspect given by the protrusion of the dorsal keel.

##### Diagnosis.

Elongate animal (~ 12 mm), light orange with a discontinuous dorsal keel with protrusions as lobes (number variable, protrusions simple or trilobed). Sclerites as hollow acicular spines, with hook-shaped and harpoon-shaped sclerites. Without epidermal papillae. Mouth and atrium partially separated. Atrium with numerous (≤20) single and branched papillae. Distichous radula. Ventrolateral foregut gland of type A / *Pararrhopalia* type. Foregut with a dorso-pharyngeal papilla gland. With 12 respiratory folds. With abdominal spicules. With one dorsoterminal sensory organ.

##### Description.

Description based on the holotype, external aspect of the paratype also considered. Reconstruction of the internal anatomy (Fig. 8B, B’) built from the manual reconstruction based on serial sections of the holotype.

**Figure 5.**
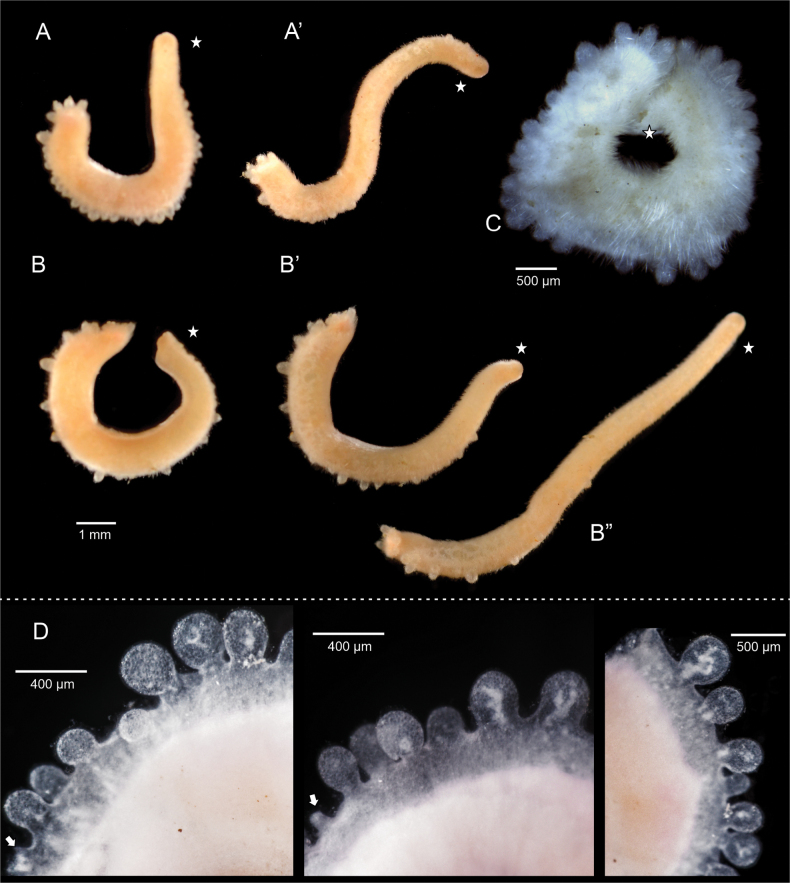
Habitus of *Eleutheromeniabullescens* sp. nov. **A, A**’ field images of the Holotype (USNM 1718004) **B, B**’ field images of the paratype (USNM 1718005) **C** paratype in 95% ethanol **D** detail of the dorsal lobes in the holotype (decalcified mid-body region). Images were captured using Olympus DSX100 optical microscope (Olympus Corporation, Tokyo, Japan) with anti-halation and fast HDR adjustments; brightness 0016 to 0022, texture 50-71, contrast 36-50. Star indicates the anterior end of the animal. Arrow indicates detached lobes and their “pedunculi.”

***Habitus*.** Elongate animal (10–12 × 0.5–1 mm), light orange in life (Fig. 5A, B), but white after preservation in ethanol (Fig. 5C). With a dorsal, discontinuous keel formed by nearly spherical lobes of different sizes. Lobes without organized arrangement, which varies between the holotype and paratype and depending on the degree of extension of the body. Lobes single or grouped as pairs or groups of three.

**Figure 6.**
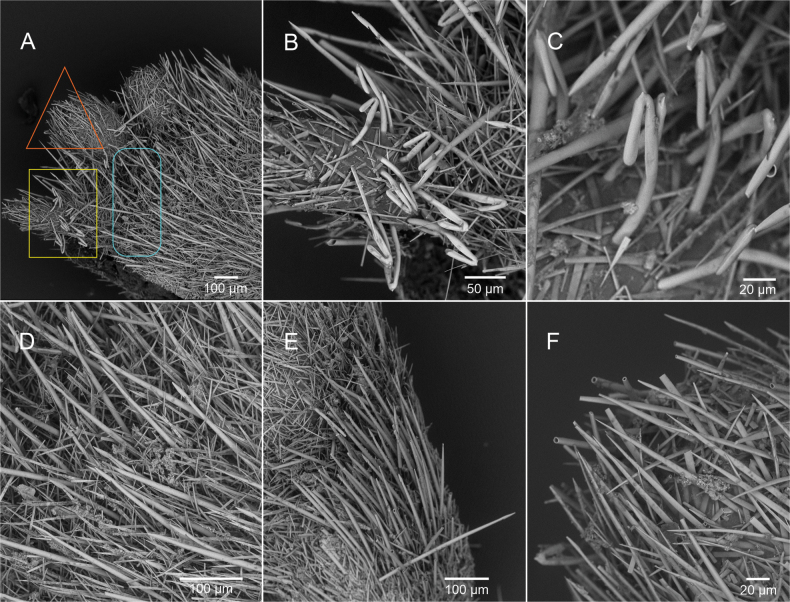
SEM images of the sclerites of *Eleutheromeniabullescens* sp. nov. **A** general view of the sclerites in the dorsal region **B** corresponds with the area in the yellow rectangle in **A** detail of sclerites **C** detail of the hook-shaped sclerites **D** corresponds with area in the blue oval rectangle in **A** detail of harpoon-like sclerites and flat acicular sclerites **E** harpoon-like sclerites in the mid-ventral body region **F** corresponds with the area in the red triangle in **A** harpoon-like sclerites, slightly curved acicular sclerites and flat acicular sclerites. (Images of the holotype: USNM 1718004).

***Mantle*.** Thin cuticle (18.31–27.6 μm) without distinct papillae and with five main types of hollow acicular sclerites protruding from it (Fig. 6): 1) Hook-shaped sclerites (Fig. 6A–C; 80–90 × 9–6 µm; the inner part of the hook is 30 µm long) with a small distal protrusion and a short internal region of the hook are particularly abundant in the dorsal region of the body and on the dorsal lobes; 2) Harpoon-shaped sclerites (Fig. 6A, D; 200–210 × 8 µm), present all over the body and are the dominant sclerite type in the mid-ventral region, also larger than elsewhere on the body (Fig. 6E; 200–300 × 8–10 µm); 3) Very thin and long acicular sclerites (Fig. 6D, E; 80–150 × 2 µm), distributed all over the surface of the body, but are less abundant in the dorsal lobes; 4) Acicular sclerites that look almost flat, but are hollow and elliptical in cross-section (Fig. 6A, D–F; 100–160 × 10 µm), present all over the body; 5) Slightly curved acicular sclerites of varying length (Fig. 6A, F; 80–160 × 6–7 µm), present all over the body. With knife-shaped scales characteristic of the pedal groove.

**Figure 7.**
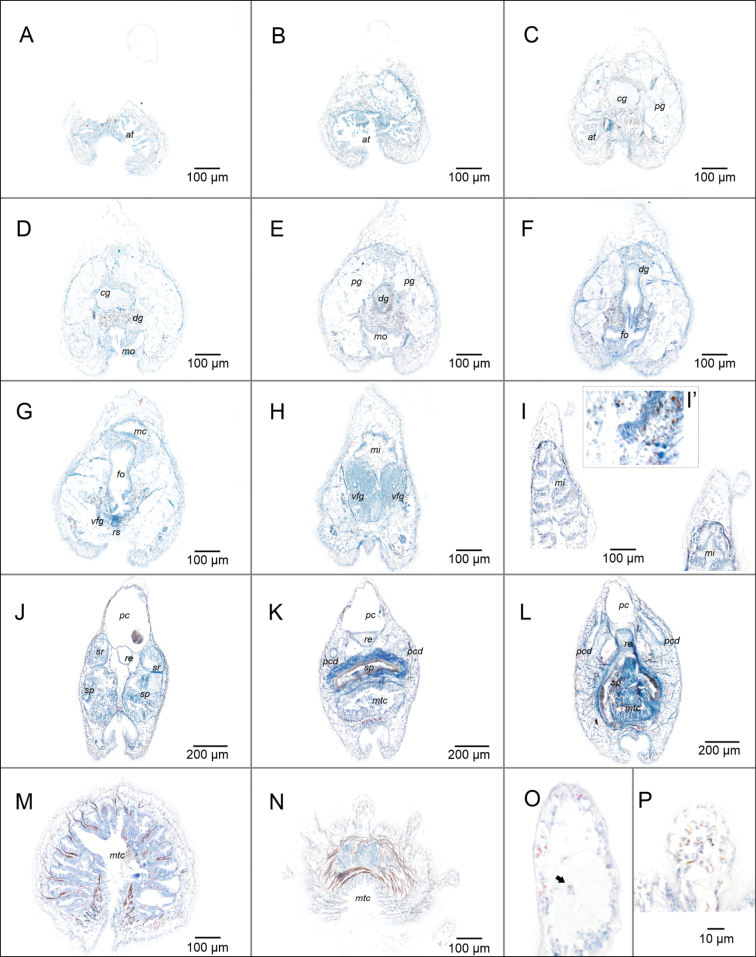
Sections of *Eleutheromeniabullescens* sp. nov. **A–G** anterior region **A–C** atrium (detail of the ventral region: muscular groove between mouth and atrium) **D, E** mouth and dorsal gland **F** foregut and dorsal gland **G** ventrolateral foregut glands, radular sac and pedal pit **H** posterior region of the ventrolateral foregut glands, midgut, and detail of the cnidocytes (**I**’) **I** midgut with constrictions **J–N** posterior region **J** paired spawning ducts, seminal vesicles and pericardioducts **K, L** fusion of the rectum, spawning ducts, and mantle cavity **M, N** respiratory folds **O, P** details of the dorsal lobes. Abbreviations: at – atrium; cg – cerebral ganglia; dg – dorsal gland; fo – foregut; mc – midgut caecum; mi – midgut; mo – mouth; mtc – mantle cavity; pcd – pericardioducts; pp –pedal pit; re – rectum; rs – radular sac; sp – spawning duct; sr – seminal receptacles; vfg – ventrolateral foregut glands. (Images of the holotype: USNM 1718004).

***Pedal groove and mantle cavity*.** Small pedal pit (90 µm long, 16 µm wide, 4–6 µm high). Pedal grove well marked, extending along the entire length of the body, with a single wide triangular pedal fold (Fig. 7H–L; 5–10 µm wide in the middle region of the fold × 5–12 µm high). Mantle cavity with 12 unbranched respiratory folds (Fig. 7M, N).

***Nervous system and sensory organs***. Cerebral ganglion of circular shape in cross section (Fig. 7B–D; 35 μm long, 20 to 22 μm wide, 10 to 14 μm high). Atrium (160 μm long, 17 to 34 μm wide, 8 to 10 μm high) with numerous (≤20) single and branched papillae (Fig. 7A, B). Without dorsoterminal sensory organ.

**Figure 8.**
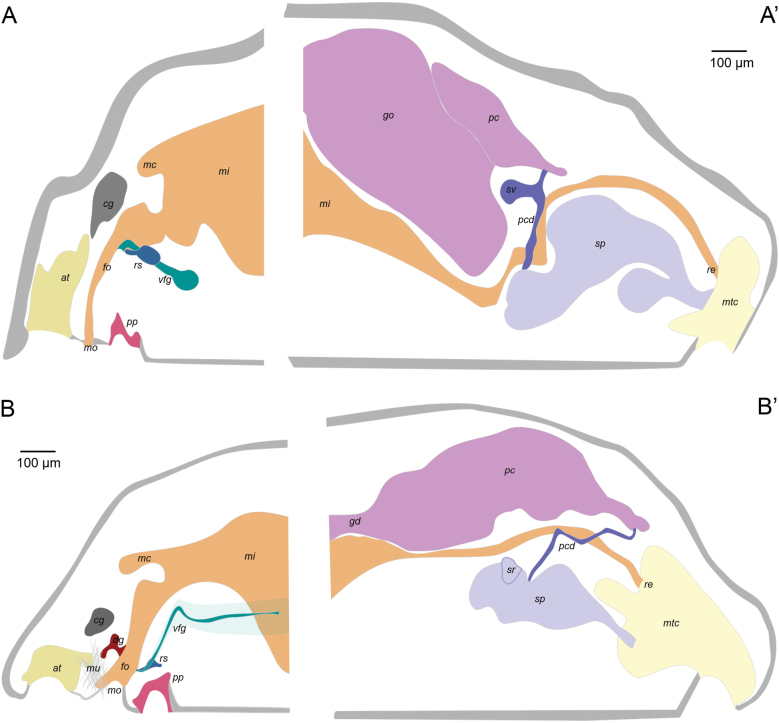
Reconstruction of the internal anatomy of **A***Dondersiatweedtae* sp. nov. **B***Eleutheromeniabullescens* sp. nov. (A anterior reconstruction, B posterior reconstruction). Abbreviations: at – atrium; cg – cerebral ganglia; dg – dorsal gland; fo – foregut; go – gonad; mu – musculature; mc – midgut caecum; mi – midgut; mo – mouth; mtc – mantle cavity; pcd – pericardioducts; pg – pedal gland; pp –pedal pit; re – rectum; rs – radular sac; sp – spawning duct; sc – seminal vesicle; sr – seminal receptacles; vfg – ventrolateral foregut glands. (Drawings based on the manual reconstruction built on the study of serial sections of the holotypes.).

***Digestive system*.** Mouth and atrium partially separated (mouth separated from the atrium by a ridge with musculature but without cuticle; Fig. 7C). Mouth (Fig. 7D) leads to a rounded foregut that enlarges dorsally, where it forms a connection with a dorso-pharyngeal papilla gland (Fig. 7D–F). Short radular sac (Figs 7G, 8B). Ventrolateral foregut glands of type-A (García-Álvarez and Salvini-Plawen 2007) / *Pararrhopalia*-type (Handl and Todt 2005 that are very glandular posteriorly (Fig. 7H). Radula distichous, formed by hook-shaped teeth (radula broken in the sections so the number of middle denticles, if present, cannot be estimated). Midgut with a dorsal caecum that projects anteriorly above the foregut and dorsal pharyngeal gland (Figs 7G, 8B) and marked lateral constrictions (Fig. 7I). Rectum ends dorsally in the mantle cavity (Fig. 7L).

***Gonopericardial system*.** Mature animal. Gonoducts connect with a large pericardium (540 µm long, 40 to 200 µm high). Heart not evident in most of the serial sections. Pericardioducts (340 μm long, 10–20 μm diameter) connect to the posterior end of the pericardium and the mid-posterior spawning duct (Figs 7J, 8B’). Spawning duct paired in most of its longitude (400 μm), ending as a single tube (160 μm long) in the middle of the mantle cavity (Fig. 7K, L). Seminal receptacles attached dorsally to each of the spawning ducts (Fig. 7J), posterior to the fusion of the pericardioducts with the spawning ducts (Fig. 8B’). Without seminal vesicles. Without copulatory stylets. With abdominal spicules.

***Anatomy of the dorsal keel*.** Dorsal keel consists of a discontinuous series of cuticular lobes. Number of lobes variable among individuals (~ 30 in the holotype and 24 in paratype 1; Fig. 5A, B). In living specimens, lobes protrude less from the cuticle when the animal expands the body (Fig. 5B’). This and the orientation of the animal makes it difficult to get an exact number of lobes. Concentration of lobes along the body seems uniform in preserved specimens (Fig. 5C), but in living specimens the density of lobes is higher in the posterior region of the body (Fig. 5A, B). This variation of the dorsal keel in the living specimens and after fixation was already described for *Eleutheromeniasierra* Pruvot, 1890 (Cobo et al. 2024). The lobes are mostly single in the mid body, but they can occur as pairs or groups of three, especially toward the anterior and posterior ends. In the decalcified animal it was evident that the lobes have a proximal peduncle in connection with the body (Fig. 5D). Both in the living and fixed specimens, and after decalcification it was observed that the lobes are easily detached from the body, breaking at the peduncular area (Fig. 5D). Histology of the dorsal keel is reminiscent of what has been described for the keel of *E.sierra* (Pruvot 1890; Salvini-Plawen 2003b: fig. 11): connection between lobes not evident externally nor in the histological series. study of the lobes under the microscope after decalcification (Fig. 5D) revealed that they contain an unidentified material that is in some way connected with the internal organs. Content not easily characterized, although diverse types of cells could be observed (Fig. 7P), including one that was tentatively identified as a cnidocyte (Fig. 7O).

##### Comparisons.

The presence of hollow sclerites with a hook-shaped distal end is characteristic of two subfamilies within the family Pruvotinidae: Pruvotininae Heath, 1911 and Eleutheromeniinae Salvini-Plawen, 1978 (García-Álvarez and Salvini-Plawen 2007). The main distinguishing feature between these subfamilies is the presence of a dorso-pharyngeal papilla gland in Pruvotininae (García-Álvarez and Salvini-Plawen 2007; Pedrouzo et al. 2022). Although several works have shown that some of the diagnostic characters of the family are somehow ambiguous and the group is in need of systematic revision (García-Álvarez and Salvini-Plawen 2007; Zamarro et al. 2013; Pedrouzo et al. 2022; Martinez-Sanjuán 2024), the presence/absence of the dorso-pharyngeal papilla gland has been considered as a good diagnostic character to distinguish these subfamilies (Cobo et al. 2024). The new species described here has a dorso-pharyngeal papilla gland, which would place it within Pruvotininae.

**Figure 9.**
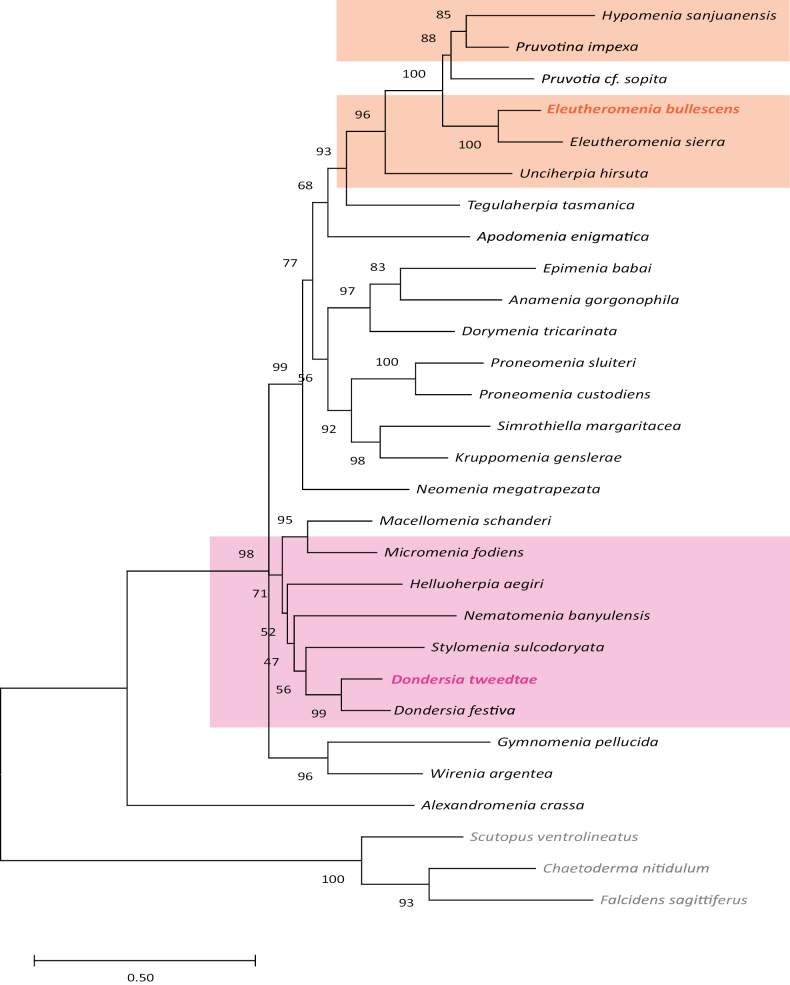
Maximum likelihood phylogenetic reconstruction based on 16S and COI genes showing the position of the new species described in this work. Bootstrap support values are shown.

The subfamily Pruvotininae includes three genera: *Pruvotina* Cockerell, 1903; *Pararrhopalia* Simroth, 1893 and *Labidoherpia* Salvini-Plawen, 1978. Traditionally, these three genera are distinguished by a combination of internal morphological characters including the presence/absence of atrio-buccal cavity (García-Álvarez and Salvini-Plawen 2007; Zamarro et al. 2013; Pedrouzo et al. 2022). Nevertheless, it was recently concluded (Cobo et al. 2024) that this is not a valid character to differentiate between genera in the subfamily Pruvotininae, and that the only apparently reliable defining morphological characteristics among these genera are the respiratory folds and copulatory stylets: respiratory folds are present both *Pruvotina* and *Labidoherpia*, but absent in *Pararrhopalia* (García-Álvarez and Salvini-Plawen 2007; Pedrouzo et al. 2022) and *Pruvotina* is the only genus in the subfamily that lacks copulatory stylets. The species described here has respiratory folds and lacks copulatory stylets and thus it would be classified as *Pruvotina*. However, the results of our phylogenetic analysis (see below and Fig. 9) place the new species as the sister taxon to a species of *Eleutheromenia* Salvini-Plawen, 1967 (Eleutheromeniinae) with maximal support and thus a classification of the new species based just on internal morphological characters is called into question.

Externally, the new species resembles *Eleutheromeniasierra* (Pruvot, 1890) due to the lobular dorsal keel (Pruvot 1890; Salvini-Plawen and Ozturk 2006; Zamarro et al. 2013; Cobo et al. 2024). Prior to the discovery of the species described here, *E.sierra* was the only species within Pruvotinidae with a distinct dorsal keel. A subtle dorsal keel has been described for *Pruvotinapeniculata* Salvini-Plawen, 1978 (Pedrouzo et al. 2022), but while a slight keel can be seen in the drawings included in the original description of the species (Salvini-Plawen 1978: figs 129, 130), this character was not apparent in our study of the sections deposited at the Smithsonian National Museum of Natural History (USNM 1604160, 1604162, 1604163, and 1604174). Further, the study of the syntypes of *P.peniculata* preserved in ethanol revealed a spiny habitus without a dorsal keel (USNM 749729). Considering the sclerites, the new species has harpoon-shaped hollow acicular sclerites, which is also a characteristic of *E.sierra* (Pruvot, 1890). This sclerite type has been reported just for one of the 16 species of *Pruvotina*, *P.harpagone* Pedrouzo, Garcia-Alvarez & Urgorri, 2022 (Pedrouzo et al. 2022). Therefore, considering the external aspect and sclerites along with the results of our molecular phylogenetic analysis, the new species is classified in the subfamily Eleutheromeniinae, despite the presence of a dorso-pharyngeal papilla gland.

Eleutheromeniinae includes two genera. No radula is present in the monospecific *Luitfriedia* García-Álvarez & Urgorri, 2001 while a distichous radula was described for the two accepted species of *Eleutheromenia*, supporting placement of the new species in this genus. *Eleutheromeniabullescens* sp. nov. can be clearly differentiated from the two known species of the genus. The dorsal keel distinguishes it clearly from *E.antarctica* (Salvini-Plawen, 1978), which lacks a keel. Despite the similarities in their external aspect, *E.bullescens* sp. nov. can be easily distinguished from *E.sierra*. The new species is orange while *E.sierra* is white to cream when alive (Pruvot 1890; Cobo et al. 2024). Moreover, the arrangement of the ≤ 30 lobes in the new species differs from what was described for *E.sierra*, which has ~ 17 single lobes, none of which are grouped, and they are spaced more regularly along the body (Cobo et al. 2024). Internally, *E.bullescens* sp. nov. has a dorso-pharyngeal papilla gland and lacks a glandular esophagus, in contrast to *E.sierra* (Salvini-Plawen 1978; Pruvot 1890; Pedrouzo et al. 2022). With the new species included here the distribution of Pruvotinidae is extended to the Gulf of Mexico (Table 3).

Considering all the above, a thorough re-evaluation of the systematics of Pruvotinidae is required. In particular, the generation and analysis of molecular data from already described species seem essential, along with a better characterization of the habitus, sclerites, radula, and digestive glands. The currently accepted classification of the family and diagnoses of the subfamilies and genera, if they prove to represent monophyletic groups, need to be amended, but more research is needed to do this adequately. Given the need for a systematic revision of Pruvotinidae, we refrain from formally amending the diagnosis of Eleutheromeniinae but note that the presence of a dorso-pharyngeal papilla gland in the new species is contrary to the current diagnosis of the group.

### ﻿DNA barcoding and phylogenetic analysis

Successful COI, 16S, and CytB sequences were obtained for both newly described species. The phylogenetic analysis performed based on COI and 16S sequences corroborated our morphology-based identification of *D.tweedtae* sp. nov. (Fig. 9), placing it as the sister taxon of *D.festiva* (bootstrap support, bs = 99). *Dondersia* was recovered within a clade of other dondersiids plus the one sampled species of Macellomeniidae, although this clade was only moderately-well supported (bs = 71).

*Eleutheromeniabullescens* sp. nov. was recovered as the sister taxon of *E.sierra* with maximal support (bs = 100). Eleutheromeniinae was recovered as the sister taxon of a clade (bs = 88) in which *Pruvotiasopita* (Pruvot, 1891) (Rhopalomeniidae Salvini-Plawen, 1978) was recovered as the sister (bs = 85) of *Pruvotinaimpexa* (Pruvot, 1890) (Pruvotinidae, Pruvotininae) and *Hypomeniasanjuanensis* Kocot & Todt, 2014 (Pruvotinidae, Lophomeniinae). Given the presence of a dorsal pharyngeal papilla gland, we had considered taxonomic assignment of *E.bullescens* sp. nov. within the genus *Pruvotina*, but results of this phylogenetic analysis support our decision to classify the new species within *Eleutheromenia*, which is also supported by the presence of a dorsal keel and harpoon-shaped sclerites. Although the goal of our analysis was to confirm our taxonomic assignment of the new species, it is noteworthy that the overall topology of the tree reconstructed based on 16S and COI is fairly consistent with recent transcriptome-based analyses of solenogaster phylogeny (Kocot et al. 2019; Yap-Chiongco et al. 2024), albeit with lower resolution and generally weaker bootstrap support values. Amphimeniidae was recovered as the sister taxon to all other sampled solenogasters with strong support (bs = 100), Neomeniidae was recovered in a clade with the other sampled members of Cavibelonia plus Lepidomeniidae and Apodomeniidae with strong support (bs = 99), and a clade including Epimeniidae, Proneomeniidae (which was recovered non-monophyletic as previously shown: Cobo et al. 2023; Yap-Chiongco et al. 2024), Strophomeniidae, and Simrothiellidae was recovered, albeit with weak bootstrap support (bs = 56).

## ﻿Discussion

### ﻿Morphological adaptations

The two species included in this study belong to distantly related families but show intriguing similarities in their external morphology, both with a lobulated keel. Nevertheless, a detailed examination of the structure of both species reveals notable differences between them. Externally, the attachment to the body and the consistency appears stronger in *D.tweedtae* sp. nov. where the keel is continuous, while in *E.bullescens* sp. nov., the lobes are not connected, and they have a more delicate appearance (they detach easily). The serial sections reveal darkly stained contents in the lobes of *D.tweedtae* sp. nov. that continues into the cuticle, suggesting a secretion or accumulative function. We did not observe anything like this in the sections of *E.bullescens* sp. nov. where the lobes contain isolated cells, and we identified at least one as a cnidocyte. Both species feed on hydrozoans, as evidenced by cnidocytes in the gut (Figs 4F’,7I’).

In the absence of a shell, mollusks adopt other defensive strategies for protection such as mimicry, crypsis, autotomy, production of defensive chemicals, or the retention of exogenous biochemically active compounds and cnidocytes from their prey (e.g., Avila 1995; Ros 1977; Wägele and Klussmann-Kolb 2005; Paul and Ritson-Williams 2008; Greenwood 2009; Neves et al. 2009; Moles et al. 2015; Goodheart et al. 2018, 2022; Winters et al. 2018; Wägele et al. 2022). Solenogastres lack a shell but are protected by a body covered by sclerites. Nevertheless, their defensive value has not been evaluated. The thickness of the cuticle and the layers and density of sclerites vary significantly among different groups. Given the lobulated keel and coloration exhibited by the species described here, in addition to their thin cuticle and sclerite cover, other defensive strategies might be hypothesized.

In *D.tweedtae* sp. nov., the nature of the dark-stained granules in the dorsal lobes is unknown. However, we speculate that the bright, contrasting coloration of this animal may represent aposematic coloration that warns would-be predators of a foul tasting, or toxic compound(s) stored in the lobes. Chemical defense has been described for many “Opisthobranchia” (reviewed in Wägele and Klussmann-Kolb 2005). In Chromodorididae Bergh, 1891 (Gastropoda, Nudibranchia) the storage of secondary metabolites occurs in dermal formations (MDFs) located in exposed parts of the mantel (usually near a distinct coloration, e.g., Carbone et al. 2013: fig. 1). The arrangement of the MDFs is specific to each chromodorid genus (Rudman 1984) and this, together with the coloration patterns, is supposed to play an important defensive role (reviewed in Carbone et al. 2013). Some chromodorid species lack typical MDFs but metabolites are still accumulated in the mantle rim (Harber et al. 2010). Further studies, including semithin sectioning or transmission electron microscopy, would be necessary to determine if the histology of the lobes of *D.tweedtae* sp. nov. can be compared with the MDFs (histology described in e.g., García-Gómez et al. 1991; Wägele and Klussmann-Kolb 2005. Besides, the chemical determination of metabolites in the tissues, and their evaluation, is also mandatory to determine putative toxicity. Aposematic coloration has also been associated with defense mechanisms related with nematocysts-based defense in Nudibranchia (Aguado and Marin 2007). Although we did find cnidocytes in the digestive system of *D.tweedtae* sp. nov., we did not find them in the bulbs of the keel. Two other species of *Dondersia* (*D.festiva* and *D.annulata*) also exhibit a bright coloration and it is known that all the species of the genus but two (whose placement in the genus is uncertain: D.?todtae and *D.foraminosa*; Klink et al. 2015; Cobo and Kocot 2021) feed on cnidarians (Salvini-Plawen 1972, 1978; Scheltema et al. 2012).

We speculate that the dorsal lobes in *E.bullescens* sp. nov. may be an adaptation analogous to those observed in nudibranchs. Some taxa within the nudibranch clade Cladobranchia are known to have the ability to sequester nematocysts (kleptocnidae) from their cnidarian prey (Edmunds 1966). The structure that houses the kleptocnidae is called a cnidosac and is located at the tips of the dorsal cerata (review within a phylogenetic context in Goodheart and Bely 2017; Goodheart et al. 2018). Again, further studies would be necessary to advance in the histological characterization of the structure of the bulbs of *E.bullescens* sp. nov. and the closely related species *E.sierra*, also known to feed on cnidarians (Pruvot 1890; Salvini-Plawen 1972).

### ﻿Species identification and taxonomic characters

In Solenogastres, the external aspect is uniform in most groups (reviewed by Cobo et al. 2023). Nevertheless, the study of the habitus is essential for the initial sorting of species within morphotypes and can be crucial in the identification and distinction between species of specific families, such as the ones described in this work, especially if images or videos of living animals are available. Live observations of solenogasters can be considered rare, and most known species have been described based on fixed material. Thus, samples like those studied here are important for a better understanding of external morphological variation in these mollusks. Here, we present two examples of solenogasters in which the external features (characterized by distinctive body protuberances and bright colorations) were useful for recognizing them as new species (*D.tweedtae* sp. nov.) or to justify their classification (*E.bullescens* sp. nov.) and will aid in their distinction and identification in the future.

Sclerites are commonly just useful for the classification of solenogasters within the four traditional orders (García-Álvarez and Salvini-Plawen 2007). Nevertheless, there are exceptions. In Dondersiidae sclerites have been shown to be useful for species delimitation (Scheltema et al. 2012; Cobo and Kocot 2021) and this is also demonstrated in this study with the description of *D.tweedtae* sp. nov. Within Pruvotinidae the reliance on sclerites alone is insufficient, but the presence/absence of hook-shaped sclerites along with some internal characteristics allows one to classify specimens to at least the subfamily level (reviewed in Pedrouzo et al. 2022). Our results support several previous works where the pivotal role of sclerites and other hard parts in solenogaster identification has been highlighted (e.g., Scheltema et al. 2012) and we agree that there is a need for detailed characterization of sclerites as they can constitute an important diagnostic character (Scheltema et al. 2012). We consider that they could be a key trait in the revision of Pruvotinidae if used in parallel with molecular data, but sequences of most described species are still unavailable and more detailed description of the sclerites of many of those is also needed.

The combination of DNA barcoding and sclerites is a promising tool for species identification (following Bergmeier et al. 2016) pending of a more complete DNA barcode library and better characterization of sclerites in most of the solenogaster groups. In this study the use of DNA barcoding is shown as a powerful tool in combination with sclerites but also considering the habitus of the species. Previous works have suggested the need for a revision of the family Pruvotinidae (García-Álvarez and Salvini-Plawen 2007; Zamarro et al. 2013; Pedrouzo et al. 2022; Martinez-Sanjuán 2024). In the present work, we include molecular evidence that supports the need for a review of the family. Moreover, with the classification of *E.bullescens* sp. nov. in *Eleutheromenia* despite having a dorso-pharyngeal papilla gland, we show that even the diagnostic characters that seemed more robust need to be reconsidered. We consider that habitus and sclerites, in combination with other traits, can be essential to solve the taxonomy of the family.

Our results recover *P.sopita* (Rhopalomeniidae) within Pruvotinidae. Considering the diagnostic characters currently accepted for Rhopalomeniidae, there is overlap with those of Pruvotinidae (García-Álvarez and Salvini-Plawen 2007): Rhopalomeniidae is supposed to lack hook-shaped sclerites, as do three subfamilies within Pruvotinidae (Lophomeniinae Salvini-Plawen, 1978; Halomeniinae Salvini-Plawen, 1978; and Unciherpiinae Garcia-Alvarez, Urgorri & Salvini-Plawen, 2001), and hook-shaped sclerites have recently been found in *P.sopita* (Cobo et al 2024); the type of radula (if present) is the same (distichous) in both families; the lack of respiratory folds established for Rhopalomeniidae is also known for Pruvotinidae (*Pararrhopalia* Simroth, 1893; *Metamenia* Thiele,1913; *Hypomenia* van Lummel, 1930, and *Forcepimenia* Salvini-Plawen, 1960) and the variety of ventrolateral foregut glands (A or C; García-Álvarez and Salvini-Plawen 2007) established within Rhopalomeniidae is also established in Pruvotinidae. Therefore, our findings warrant additional research using more conserved molecular markers to enhance our understanding of the relative phylogenetic placement of these families. Furthermore, a thoughtful review of the morphological characters and their significance is needed.

### ﻿New insights from the Gulf of Mexico using ARMS

Autonomous Reef Monitoring Structures (ARMS) are shown here, as in previous works, as a useful tool for biodiversity assessment and characterizing cryptic biodiversity (e.g., Brainard et al. 2009; Ransome et al. 2017; Hazeri et al. 2019; Vital et al. 2023). Moreover, this study highlights their use for live observations of relatively small and difficult-to-find taxa such as solenogasters, and thus their role in advancing the taxonomy and ecological knowledge. The findings of this study provide new data on the distribution of species within Dondersiidae and Pruvotinidae (Table 3).

To date, only two species of solenogasters from the Gulf had been formally described: *Proneomeniaacuminata* Wirén, 1892, originally described from the Antilles and later recorded in the Florida Channel (Wirén 1892; Heath 1911) and *Spengelomeniabathybia* Heath, 1912 described from a specimen found among “a small collection of alcyonarian corals that had been secured from a cable ship operating to the Northwest of the Florida” (Heath 1912: 30). Besides these two species, the aplacophoran fauna of the Gulf of Mexico was documented in 1979 with the additional record of 134 specimens of unnamed Caudofoveata (Treece 1979).

Since then, eight Caudofoveata species have been formally described (*Chaetodermafelderi* Scheltema & Ivanov, 2007; *Chevrodermacuspidatum*, *Clavidermaamplum*, *Spathodermabulbosum*, *Clavidermamexicanum*, *Prochaetodermagilrowei*, *Niteomicacaptainkiddae* and *Spathodermaquadratum*; Ivanov and Scheltema 2008) and numerous other specimens, including several unnamed Solenogastres, have been collected and are held in scientific collections, particularly at the Smithsonian National Museum of Natural History. Despite the substantial number of available specimens most solenogaster species from the Gulf of Mexico remain undescribed.

The Gulf of Mexico (GOM) faces significant anthropogenic pressures, notably from coastal human activities, the Mississippi River discharge, and the oil industry (McKinney et al. 2021). Moreover, although the GOM is considered a well-studied region, new species from various taxa, specially neglected small-bodied invertebrates, continue to be discovered (e.g., Hernández-Alcántara and Solís-Weiss 2000; Järnegren et al. 2007; Opresko et al. 2020; Ortiz and Cházaro-Olvera 2022, 2024). To protect this area and to create conservation figures, addressing gaps in biodiversity knowledge is essential. The two species included in this work (*D.tweedtae* sp. nov. and *E.bullescens* sp. nov.) constitute an example of these efforts.

## ﻿Conclusions

The findings reported here underscore the importance of ARMS as a sampling method to collect rare taxa and of integrative taxonomic approaches including the study and observation of living specimens. The identification of these remarkable new species offers fresh insights into the diversity, systematics, morphological variety, and ecology of the group. The obtained molecular data contributes to a growing database for solenogasters which is helping to accelerate the process of identification and species discovery, and advance understanding relationships within the group. However, available data for the group remains limited and continued work is necessary to represent much of its diversity. This research also marks a step forward in understanding the real diversity of Solenogastres from the Gulf of Mexico.

## Supplementary Material

XML Treatment for Dondersia

XML Treatment for Dondersia tweedtae

XML Treatment for Eleutheromenia

XML Treatment for Eleutheromenia bullescens

## ﻿Additional information

### Author ORCIDs

M. Carmen Cobo https://orcid.org/0000-0002-8481-2086

William J. Farris https://orcid.org/0009-0001-8687-6657

Chandler J. Olson https://orcid.org/0000-0002-1524-1339

Emily L. McLaughlin https://orcid.org/0009-0006-0297-2067

Kevin M. Kocot https://orcid.org/0000-0002-8673-2688

### Data availability

All specimens are deposited in the collections of the National Museum of Natural History (NMNH), Smithsonian Institution, Washington DC. Specimens’ occurrences are included in GBIF via the NMNH Extant Specimen Records dataset (https://www.gbif.org/dataset/821cc27a-e3bb-4bc5-ac34-89ada245069d). Generated sequences are deposited in the National Center for Biotechnology Information (https://www.ncbi.nlm.nih.gov/). This article is registered in ZooBank under https://zoobank.org/C48FCB58-0A35-4D73-9EA1-D9943395ED0B.
